# Volume of red blood cell transfusion is a risk factor for acute ischemic stroke after non-variceal upper gastrointestinal bleeding

**DOI:** 10.3389/fneur.2025.1651661

**Published:** 2025-09-19

**Authors:** Fan Zhou, Jiaming Huang, Donghua Huang

**Affiliations:** Ganzhou People’s Hospital, Ganzhou, China

**Keywords:** acute ischemic stroke, non-variceal upper gastrointestinal bleeding, transfusion, red blood cell, risk factor

## Abstract

**Objective:**

To determine if the volume of red blood cell (RBC) transfusion is a risk factor for acute ischemic stroke (AIS) in patients with non-variceal upper gastrointestinal bleeding (NVUGIB).

**Methods:**

NVUGIB patients who received RBC transfusion between January 2019 and December 2021 were included. Patients were divided into two groups, AIS group and non-AIS group. Propensity score matching was used to match the confounding factors between the two groups. Multivariate logistic regression was used to analyze independent risk factor for AIS in NVUGIB patients. The ROC curve was used to assess the sensitivity and specificity of the factors.

**Results:**

The study included 272 NVUGIB patients who received RBC transfusions, with 44 patients in the AIS group and 228 patients in the non-AIS group. After propensity score matching, 38 pairs of patients were successfully matched. Multivariate logistic regression showed that the volume of RBC transfusion was an independent risk factor for AIS in NVUGIB patients. The area under the curve for RBC transfusion volume was 0.811, the sensitivity and specificity were 0.553 and 0.921, respectively.

**Conclusion:**

The volume of RBC transfusion is a risk factor for AIS in NVUGIB patients. However, these results need to be validated by multicenter, prospective studies with larger sample sizes.

## 1 Introduction

Red blood cell (RBC) transfusion is commonly used in clinical practice to treat anemia. However, RBC transfusions are not always associated with positive outcomes. A study found that perioperative transfusion of red blood cell during heart valve surgery was associated with ischemic stroke (1). Similarly, for those patients who underwent lung transplantation, transfusion of red blood cell before surgery was associated with increased morbidity and mortality (2). Additionally, perioperative transfusion of packed red blood cell was associated with an increased mortality risk in those patients who underwent surgery for cancer (3). A study by Kim et al. (4) found that late red blood cell transfusion was a predictor for poor outcome in patients with AIS. Our previous study found that RBC transfusion was a risk factor for acute cerebral infarction (AIS) in non-variceal upper gastrointestinal bleeding (NVUGIB) patients (5). However, the reason why RBC transfusion increases the risk of AIS in NVUGIB patients remains unclear. It was found that the number of transfused RBC units was an independent predictor of stroke after transcatheter aortic valve replacement. A study in Queensland supported that RBC transfusion was associated with adverce outcome in a dose-dependent manner (6). So, is volume of RBC transfusion an explanation of increased risk of AIS in NVUGIB patients? Therefore, we retrospectively analyzed the data from NVUGIB patients admitted to Nanchang University Affiliated Ganzhou Hospital to investigate this issue.

## 2 Patients and methods

### 2.1 Study design and patients

NVUGIB patients who received RBC transfusion between January 2019 and December 2021 were included. NVUGIB was defined as non-variceal gastrointestinal bleeding occurring above Treitz’s ligament (7). The primary symptoms of NVUGIB included hematemesis, coffee-ground vomiting, and melena.

The inclusion criteria were as follows: (1) patients with hematemesis and/or melena or a positive stool occult blood test; (2) patients diagnosed with NVUGIB by endoscopy; (3) patients who received RBC transfusions. The clinical diagnosis and endoscopic diagnosis were made according to the diagnostic criteria of NVUGIB (8, 9). The exclusion criteria were as follows: (1) patients who had AIS within 1 month before NVUGIB; (2) AIS patients who were not confirmed by brain CT or MRI; and (3) patients with incomplete data.

### 2.2 Patient classification and treatment

Patients were divided into two groups, AIS group and non-AIS group. Patients in the AIS group were complicated with AIS, and patients in the non-AIS group were not. The definition of AIS was rapidly occurring neurological impairment caused by brain ischemia, confirmed by brain CT or MRI. The diagnosis of AIS was confirmed by two senior physicians. All patients were treated according to NVUGIB treatment guidelines (8). Patients underwent endoscopic examinations, received red blood cell transfusions, fluid supplementation and intravenous proton pump inhibitors (PPIs). Additional treatments included hemagglutinin, octreotide, somatostatin, and mechanical ventilation as needed.

Data of the patients were collected from the hospital’s electronic medical record system, including: (1) age and sex; (2) smoking and drinking; (3) medical history: hypertension, coronary heart disease (CHD), diabetes, previous cerebral infarction, gout, peptic ulcer, chronic obstructive pulmonary disease (COPD) and chronic kidney disease (CKD); (4) treatment: mechanical ventilation; (5) RBC data: volume of RBC transfusion, duration of RBC storage.

Blood samples were taken within 24 h after admission for routine blood tests, liver function tests, kidney function tests and coagulation function tests. Data such as white blood cell (WBC), hemoglobin, red blood cell distribution width (RBC-DW), platelet, mean platelet volume (MPV), nitrogen, creatinine, prothrombin time (PT), fibrinogen, activated partial thromboplastin time (APTT), D-dimer, total bilirubin and albumin were collected.

### 2.3 Red blood cell transfusion

In our hospital, on average, one unit of RBC is about 150 mL. The RBC is stored in CPDA solution (citrate–phosphate-glucose-adenosine solution). Leukoreduced RBCs were transfused. RBCs were not irradiated.

### 2.4 Propensity score matching

Propensity score matching was used to match confounding factors between the two groups. Complication of AIS was used as the dependent variable, sex, age, smoking, drinking, hypertension, CHD, diabetes, previous cerebral infarction, gout, peptic ulcer, CKD, COPD and mechanical ventilation were used as the independent variables, logistic regression was used to calculate the propensity score for each patient. Complete matching and random arrangement of cases were used as the matching method.

### 2.5 Ethics approval and registration

This retrospective clinical study was approved by the Ethics Committee of Nanchang University Affiliated Ganzhou Hospital. The study was conducted in accordance with the Declaration of Helsinki. Informed consent was obtained from all patients.

The study was registered in Medical Research Registration and Filing Information System (No. was MR-36-24-047923).

### 2.6 Statistical analysis

SPSS26.0 software was used for statistical analysis. Continuous variables were expressed as mean ± standard deviation, categorical variables as frequencies. The student’s *t*-test or Mann–Whitney test was used for continuous variables if appropriate, and the chi-square test for categorical variables. Propensity score matching was used to match the confounding factors. Univariate regression analysis was used to select factors for multivariate logistic regression, and factors with a *p* < 0.1 in the univariate analysis were included. ROC curve was used to assess the sensitivity and specificity of the factors. *p* < 0.05 was considered statistically significant.

## 3 Results

### 3.1 Cohort characteristics

The study included 272 NVUGIB patients who received RBC transfusions, with 44 patients in the AIS group and 228 in the non-AIS group. Among them, 203 patients were male, 153 patients were older than 60 years (Table 1).

**Table 1:** Baseline characteristics of patients before propensity score matching.

Factors	AIS group (*n* = 44)	Non-AIS group (*n* = 228)	*p*-value
Sex (male)	33	170	0.951
Age (>60 years old)	35	118	0.001
Smoking	7	60	0.142
Drinking	6	56	0.114
Hypertension	32	84	0.000
CHD	7	18	0.092
Diabetes	14	19	0.000
Previous cerebral infarction	9	16	0.005
Gout	3	22	0.552
CKD	2	19	0.389
Peptic ulcer	6	46	0.313
COPD	2	8	0.738
Mechanical ventilation	18	57	0.031

The AIS group had more patients over 60 years old, with higher rates of hypertension, diabetes, previous cerebral infarction, and mechanical ventilation. There was no significant difference in terms of sex, smoking, drinking, CHD, gout, CKD, peptic ulcer and COPD between the two groups.

### 3.2 Propensity score matching

To match the baseline characteristics between the two groups, we performed propensity score matching. Sex, age, smoking, drinking, hypertension, CHD, diabetes, previous cerebral infarction, gout, CKD, peptic ulcer, COPD and mechanical ventilation were used as factors for PSM, and 38 pairs of patients were successfully matched. After matching, there was no significant difference in baseline characteristics between the two groups (Table 2).

**Table 2:** Baseline characteristics of patients after propensity score matching.

Factors	AIS group (*n* = 38)	Non-AIS group (*n* = 38)	*p*-value
Sex (male)	28	27	0.798
Age (>60 years old)	29	32	0.387
Smoking	7	12	0.185
Drinking	6	7	0.761
Hypertension	26	26	1.000
CHD	5	5	1.000
Diabetes	9	6	0.387
Previous cerebral infarction	7	3	0.175
Gout	2	3	0.644
CKD	2	0	0.152
Peptic ulcer	6	6	1.000
COPD	2	1	0.556
Mechanical ventilation	14	8	0.129

### 3.3 Comparison of laboratory results and RBC transfusion data after matching

After matching, hemoglobin levels were significantly lower in the AIS group, while D-dimer and albumin levels were significantly higher in the AIS group than non-AIS group, patients in the AIS group also received more RBC transfusions than the non-AIS group (Table 3).

**Table 3:** Laboratory tests and RBC transfusion data of patients after propensity score matching.

Factors	AIS (*n* = 38)	Non-AIS (*n* = 38)	*p*-value
WBC (10^9^/L)	9.89 ± 4.66	8.83 ± 4.01	0.292
Hemoglobin (g/L)	59.92 ± 10.62	69.03 ± 20.71	0.018
RBC-DW	15.97 ± 3.72	15.66 ± 3.89	0.721
Platelet (10^9^ /L)	190.39 ± 95.12	198.21 ± 101.23	0.730
MPV (fL)	9.34 ± 1.31	9.96 ± 1.63	0.073
Nitrogen (mg/L)	12.55 ± 6.52	12.88 ± 9.55	0.860
Creatinine (μmol/L)	154.76 ± 130.00	119.11 ± 109.54	0.200
PT(s)	12.48 ± 1.32	13.13 ± 4.93	0.437
Fibrinogen (g/L)	2.35 ± 1.46	2.22 ± 0.97	0.643
APTT(s)	27.60 ± 8.10	26.21 ± 6.16	0.403
D-dimer	2.79 ± 3.40	1.26 ± 1.66	0.015
Ttotal bilirubin (μmol/L)	10.20 ± 6.37	11.02 ± 10.41	0.680
Albumin (g/L)	31.56 ± 5.35	28.66 ± 4.82	0.015
Volume of RBC transfusion (unit)	5.32 ± 2.76	2.74 ± 2.03	<0.001
Duration of RBC storage (day)	15.74 ± 6.30	15.09 ± 6.77	0.667

### 3.4 Univariate logistic regression analysis of risk factors

Univariate logistic regression showed D-dimer and RBC transfusion volume were risk factors for AIS in NVUGIB patients, hemoglobin and albumin were protective factors (Table 4).

**Table 4:** Univariate logistic regression analysis of risk factors.

Factors	*β*	OR	95% confidence interval for OR	*p*-value
WBC	0.061	1.063	0.947, 1.193	0.302
Hemoglobin	−0.036	0.964	0.934, 0.996	0.026
RBC-DW	0.022	1.022	0.907, 1.153	0.717
Platelet	−0.001	0.999	0.995, 1.004	0.726
MPV	−0.295	0.745	0.535, 1.036	0.080
Nitrogen	−0.005	0.995	0.941, 1.052	0.857
Creatinine	0.003	1.003	0.998, 1.007	0.218
PT	−0.061	0.940	0.794, 1.113	0.475
Fibrinogen	0.089	1.093	0.755, 1.581	0.638
APTT	0.028	1.029	0.963, 1.099	0.403
D-dimer	0.266	1.304	1.028, 1.655	0.029
Total bilirubin	−0.011	0.989	0.937, 1.043	0.677
Albumin	−0.115	0.891	0.809, 0.982	0.020
Volume of RBC transfusion	0.495	1.640	1.251, 2.151	<0.001
Duration of RBC storage	0.016	1.016	0.947, 1.089	0.663

### 3.5 Multivariate logistic regression analysis of risk factors

Factors with a *p*-value < 0.1 in the univariate logistic regression were used for multi-variate logistic regression analysis, it showed RBC transfusion volume was an independent risk factor for AIS in NVUGIB patients (Table 5).

**Table 5:** Multivariate logistic regression analysis of risk factors.

Factors	*β*	OR	95% confidence interval for OR	*p*-value
Volume of RBC transfusion	0.432	1.54	1.176, 2.016	0.002
Hemoglobin	−0.021	0.979	0.944, 1.015	0.251
MPV	−0.433	0.649	0.409, 1.027	0.065
Albumin	−0.066	0.936	0.832, 1.053	0.272
D-dimer	0.166	1.181	0.899, 1.552	0.232

### 3.6 ROC curve assessing the sensitivity, specificity and cut-off value of RBC transfusion volume

**Figure 1:**
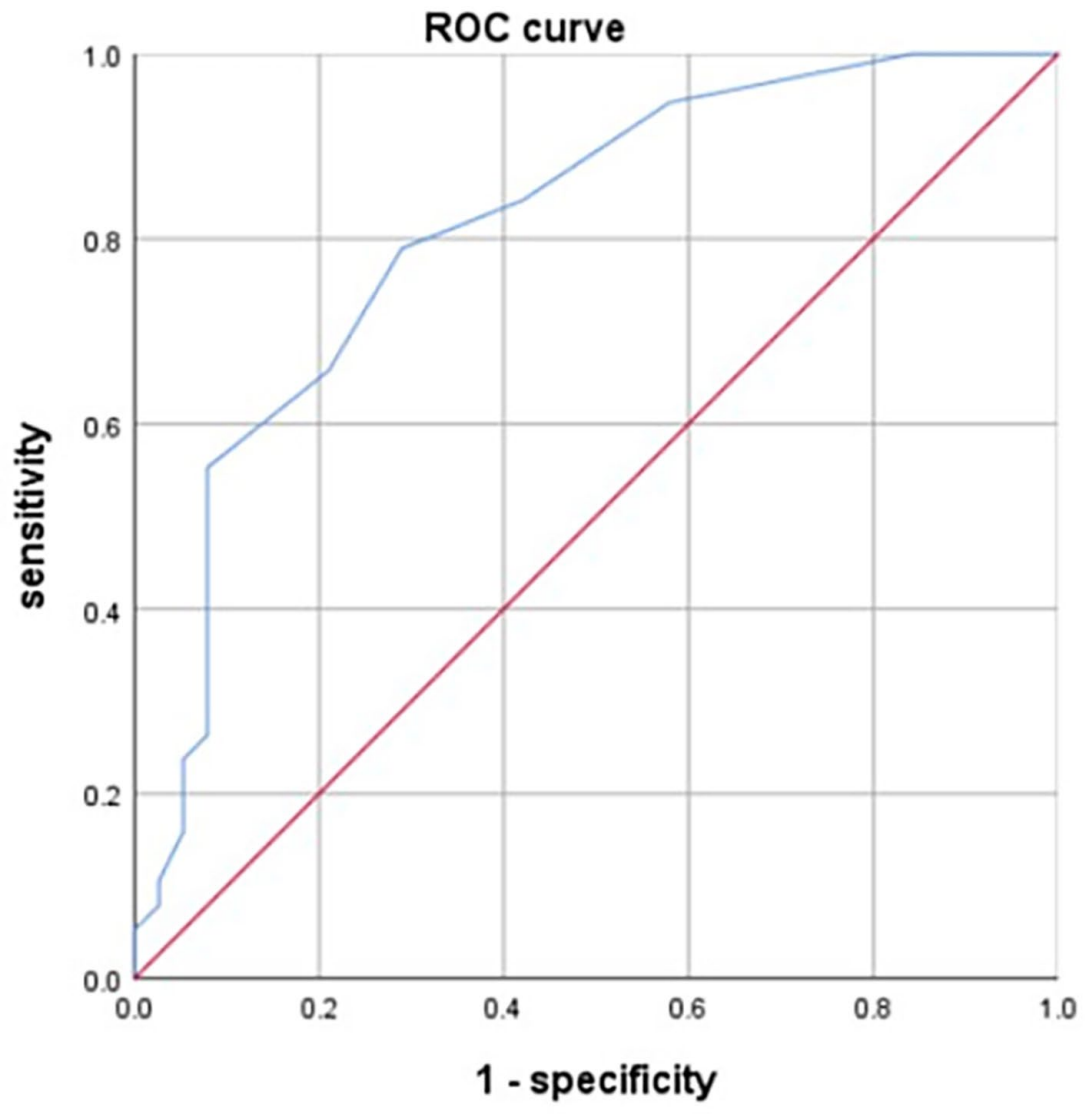
ROC curve assessing the value of RBC transfusion volume.

The sensitivity, specificity and cut-off value of RBC transfusion volume for AIS in NVUGIB patients was determined by ROC curve. The area under curve (AUC) was 0.811, the sensitivity and specificity of RBC transfusion volume were 0.553 and 0.921, respectively. The optimal cut-off value of RBC transfusion volume was 4.5 units (Figure 1).

## 4 Discussion

NVUGIB is a common gastrointestinal condition that can lead to severe complications such as shock, acute renal injury, acute myocardial infarction, and ischemic stroke (10, 11). Our previous study found that the incidence of AIS in NVUGIB patients was around 5.38% (5).

RBC transfusions are often necessary for severe NVUGIB patients, but they can also lead to adverse outcomes. Studies have shown that RBC transfusions increase the risk of mortality in surgical patients (3) and are associated with AIS in NVUGIB patients (5), as well as a predictor for poor outcome in patients with AIS (4). Volume of RBC transfusion has profound effects on patients. Yuan found that volume of RBC transfusion was closely related with wound infection in surgery patients (12). Another study found that the more units of RBC patients received, the higher risk of morbidity, acute kidney injury and mechanical ventilation patients had (13). A study in Queensland divided cardiac surgery patients into two groups, one group of patients who received transfusion ≥5 units of packed RBC, and another group of patients who received transfusion ≤4 units of packed RBC, the results showed patients received transfusion ≥5 units of packed RBC had higher rate of in-hospital mortality, acute renal failure, acute myocardial infarction and stroke complications (6). Similarly, our study found that the volume of RBC transfusion was a risk factor for AIS in NVUGIB patients, with an optimal cut-off value of 4.5 units.

During storage, red blood cell makes numerous biochemical, structural, inflammatory, and physiologic changes. These changes might have profound clinical effects on patients. But, the duration of RBC storage has been debated as a potential cause of complications. Vamvakas and Carven (14) reported a statistical correlation between the length of storage of the RBCs and the development of pneumonia or wound infection. Leal-Noval et al. (15) found that duration of storage of RBCs was not correlated to the development of myocardial infarction or pneumonia. Obonyo, et al. (6) also found that duration was storage of RBCs was not associated with significantly increased mortality or morbidity. A study compared patients who received transfusion of red blood cells stored for 10 days or less with those patients who received transfusion of red blood cells stored for 21 days or more, the results showed no significant differences in the complications between the two groups (16). In this study, we analyzed the duration of RBC storage on the development of AIS in NVGUIB patients, and found that there was no significant difference in the duration of RBC storage between the two groups.

### 4.1 Limitations

This study has some limitations. First, this was a single-center, retrospective study. Second, the number of patients included in this study was relatively small. Third, the sensitivity, specificity and cut-off value of RBC transfusion volume were validated using ROC curve, but, not validated using external data.

In conclusion, volume of RBC transfusion is a risk factor for AIS in NVUGIB patients. However, the result still needs to be validated by multi-center, prospective study with larger sample size.

## Data Availability

The original contributions presented in the study are included in the article/supplementary material, further inquiries can be directed to the corresponding author.
